# Does Parental Media Soothing Lead to the Risk of Callous–Unemotional Behaviors in Early Childhood? Testing a Moderated Mediation Model

**DOI:** 10.3390/bs15081082

**Published:** 2025-08-08

**Authors:** Ruifeng Tan, Kai Hu, Peishan Huang, Liman Cai

**Affiliations:** 1 School of Education, South China Normal University, Guangzhou 510631, China; 2 School of Education, Guangzhou University, Guangzhou 510006, China; 3 School of Education (Shanwei), South China Normal University, Shanwei 516622, China; 4 Faculty of Preschool Education and Humanities, Dongguan Polytechnic, Dongguan 523808, China; 5 Center for Studies of Psychological Application, South China Normal University, Guangzhou 510631, China

**Keywords:** parental media soothing, emotion regulation, callous–unemotional behaviors, effortful control

## Abstract

Callous–unemotional (CU) behaviors are a significant marker of early socioemotional disorders. This study investigated the role of parental media soothing as a potential risk factor for CU behaviors in young children and the indirect effects of children’s emotion regulation competence and effortful control. Data were collected from 1095 Chinese parents of young children (M_age(mouths)_ = 60.56, SD = 9.52) using the Media Emotion Regulation Scale, the Emotion Regulation Scale, the Inventory of Callous–Unemotional Traits, and the Effortful Control Scale. Moderated mediation analysis was employed to examine whether parental media soothing indirectly impacts CU behaviors by decreasing emotion regulation and whether this indirect effect was influenced by children’s effortful control. The results were consistent with the moderated mediation model, indicating that media soothing significantly correlates with higher levels of CU behaviors through lower levels of emotion regulation. Furthermore, the indirect effect could be influenced by the level of effortful control. The findings highlighted the new familial ecological risk factors associated with early CU behaviors and provided direction for future research on the association between Chinese parental media practices and poor socioemotional outcomes in early childhood.

## 1. Introduction

Early socioemotional development is crucial for future academic and career success, as it can reduce antisocial behavior and promote interpersonal cooperation relationships (OECD, 2015) and support broader mental health by lowering the risks of childhood externalizing disorders and anxiety (Thomson et al., 2019). Conversely, deficits in early prosocial emotions are of particular concern due to the costs involved and harm to social interactions (Hepach & Vaish, 2020; Sznycer et al., 2021). For instance, callous–unemotional (CU) behaviors might occur frequently in preschool children (Waller & Hyde, 2017) and have been identified as one of the symptoms of socioemotional development deficits and externalizing symptoms in early childhood, including Oppositional Defiant Disorder and Conduct Disorder (Zumbach et al., 2021; Ritchie et al., 2022; Tan et al., 2023).

Notably, parental practices, particularly parental emotion socialization, are pivotal in shaping young children’s socioemotional functioning (Carapito et al., 2020) and might even influence CU behaviors. Unlike general parenting styles, some parents are increasingly using digital devices to regulate their children’s emotions (Dalope & Woods, 2018; Coyne et al., 2021; Radesky et al., 2016). This new parental practice of delegating children’s emotional skills to electronic devices may pose developmental risks to young children’s socioemotional functioning (Kim-Spoon et al., 2013; Sala et al., 2014), although it may seem effective in alleviating the negative emotional state of children in the short term. Children who rely on external media-based strategies for emotional management may develop deficits in adaptive self-regulation (Gordon-Hacker & Gueron-Sela, 2020; Radesky et al., 2023; Konok et al., 2024), which in turn are strongly associated with the emergence of early CU reactions (B. Liu et al., 2022; Wilke & Goagoses, 2023).

Given its developmental significance, identifying modifiable environmental and mechanistic influences on CU behaviors has become a priority in developmental psychopathology research (White & Frick, 2010). Therefore, the effects and indirect paths of parental emotion socialization based on digital media regarding early CU behaviors require exploration.

### 1.1. Parental Media Soothing and Children’s CU Behaviors

In general, CU behaviors are characterized by behavioral tendencies of low empathy, remorse, and guilt in early childhood (Waller & Hyde, 2018), followed by indifference toward academic achievements, social relationships, and reactions to rewards and punishments (Frick, 2009; Ciucci et al., 2014). Children with high levels of CU behaviors tend to be less emotionally responsive (Kimonis et al., 2006) and show coldness to others’ emotions, such as fear (Dadds et al., 2008; Marsh et al., 2011). Early CU behaviors are considered robust factors of long-term adverse outcomes, including aggression, externalizing problem behaviors, and impaired psychosocial functioning (Waller et al., 2015; Ritchie et al., 2022; Tan et al., 2023). Notably, CU behaviors may also be adaptive outcomes of exposure to adverse parenting practices (S. G. Craig et al., 2021), particularly those involving maltreatment, harsh parenting, neglect, and limited parental warmth and involvement (Roman et al., 2024; Zhu et al., 2024).

Parental practices related to emotion socialization (e.g., parental emotion-focused reactions) directly shape children’s emotional and behavioral development (Mirabile et al., 2018; Di Giunta et al., 2021). For example, parental media soothing (PMS), or media emotion regulation, refers to parenting styles that use media to regulate children’s emotional lability and negativity (Radesky et al., 2016). A few empirical studies have shown that parental media soothing is significantly linked to poor socioemotional outcomes and externalizing problem behaviors in early childhood (Coyne et al., 2022, 2023). Specifically, media engagement in parenting might hinder parent–child interactions and undermine parental warmth (Stockdale et al., 2018). Children with lower levels of parental warmth tend to show an indifferent attitude and limited prosocial emotions in daily life (Waller et al., 2014; Zheng et al., 2024)—the prominent features of CU behaviors. When parents pass their children’s emotional management to digital devices, children who habitually use media to cope with emotional distress exhibit difficulty disengaging from devices (Coyne et al., 2022). This further implies that screen time replaces face-to-face interaction, thus impairing children’s socioemotional functioning (Skalická et al., 2019; W. Liu et al., 2021).

Parents might adopt the parenting strategies of media-based emotional soothing; in other words, they may forgo parental warmth and positive emotional socialization due to digital media, thus increasing the risk of early childhood CU behaviors.

### 1.2. Children’s Emotion Regulation as the Mediator

Early childhood CU behaviors might be closely associated with certain proximal and developmental factors, particularly children’s emotional skills. Emotion regulation (ER) refers to the personal ability of monitoring, evaluating, and adjusting emotional responses to achieve specific goals (Gross, 2014). The process of regulating emotions may operate both explicitly and implicitly, involving complex interactions between cognition skills and emotion reactivity (Braunstein et al., 2017). Based on the tripartite model of the impact of the family on children’s emotion regulation and adjustment (Morris et al., 2007), the effect of parental factors on children’s emotion regulation operates through three paths, involving children’s observation of parental emotion regulation performance, emotion-related parenting practices, and the emotional climate of the family. For example, parents could provide assistance in improving children’s emotional management and adjustment skills through parenting practices such as emotional coaching (Morris et al., 2017), positive and timely responses to children’s emotions (Rutherford et al., 2015), and building a warm family emotional atmosphere (Morelen et al., 2016).

However, parental media soothing might not reach the desired effect. Although the distraction of attention from stressful stimuli or negative emotions can be efficient to reduce the intensity of children’s emotions (Davis et al., 2016), parental media soothing might strengthen early childhood media dependence and weaken the adaptive self-regulation competence of young children (Radesky et al., 2014; Coyne et al., 2022; Radesky et al., 2023). Specifically, media soothing might not enable parents to provide emotional coaching and proper emotional responses for young children and establish a positive family emotional atmosphere. For instance, maternal media soothing could correlate to children’s elevated negative emotions (Gordon-Hacker & Gueron-Sela, 2020). Moreover, existing studies examined that most children exceed the recommended media use time (Christakis, 2014; Radesky et al., 2020; Uzundağ et al., 2022), which might imply that children may increasingly rely on digital media rather than parent–child interactions for emotional regulation needs.

Furthermore, prior studies have shown that children with poor emotion regulation generally exhibit heightened CU behaviors (Graziano et al., 2022; A. G. Craig et al., 2025). Emotion dysregulation may diminish self-experienced guilt, which in turn increases the risk of CU behaviors (Manole & Enea, 2024). Specifically, emotion regulation is key for empathy. Higher emotion regulation means fewer self-focused emotions, likely allowing individuals to be more considerate of and supportive toward others’ emotions (Eisenberg, 2000). Research on preschoolers showed that emotion regulation mediates the relation between attachment security and empathy (Ştefan & Avram, 2018), which could imply that secure attachment enhances awareness of emotion regulation in early childhood, thereby boosting empathy levels of children. Previous literature showed that family media engagement in parenting was negatively correlated with parent–child attachment (Santana-Vega et al., 2019; Hood et al., 2021; Linder et al., 2021). It could be inferred that parental media soothing may likewise weaken children’s emotion regulation, reduce their empathy and guilt, and increase the risk of CU behaviors.

### 1.3. Children’s Effortful Control as the Moderator

Effortful control (EC) typically refers to the temperamental aspects of attentional and inhibitory control, involving self-control of cognitive resources as well as of impulses or behavioral tendencies (Vroman & Durbin, 2015). Based on generally temperamental theories of risk, children with high effortful control could regulate negative emotions through effective attention strategies (Zhang et al., 2023; Kochanska & Knaack, 2003). For example, children with higher effortful control would exhibit greater emotional stability, better task persistence, and more adaptive coping strategies (Kochanska & Kim, 2013; Zeytinoglu et al., 2017). In addition, children with higher effortful control would be protected against the negative effects of contextual risks, exhibiting low levels of emotional reactivity and maladaptation (Thompson et al., 2020; Lengua et al., 2008). Thus, effortful control would serve as the protector for adaptive emotion regulation in early childhood.

Specifically, the risk-protective model proposed that intrinsic protective factors could buffer and weaken the adverse effects of external risk factors on children’s mental development (Werner, 2000; Hollister-Wagner et al., 2001). Children with higher effortful control might conduct flexible and effective coping styles to regulate the negative emotions related to contextual risk factors in the family. For instance, a longitudinal study indicated that higher effortful control was linked to decreased anxiety and depression reactivity of children, even within negative parenting contexts (Kiff et al., 2011). Children with lower effortful control might not be protected against the negative effects of parental media soothing (Arık, 2024; Fitzpatrick et al., 2022; Danet et al., 2022; Radesky et al., 2023), struggle to disengage from screen-based regulation, and fail to acquire adaptive emotion self-regulation skills (Scott & Hakim-Larson, 2021; Konok et al., 2024).

Additionally, according to the protective–protective model (Fergus & Zimmerman, 2005), the effect of one protective factor on the outcome variable would be enhanced or weakened by another protective factor (Chen et al., 2015). Specifically, the facilitation hypothesis proposes that the two protective factors enhance each other, while the exclusion hypothesis suggests that one protective factor diminishes the effect of the other on the outcome variables (Chen et al., 2015). For instance, the previous study has demonstrated that higher effortful control is generally associated with a lower degree of CU behaviors in early childhood (Winebrake et al., 2024). Another study indicated the interaction of effortful control and negative emotionality likely impacts children’s behavioral adjustment, particularly behavioral symptoms related to externalizing problems (Wilson et al., 2021). Accordingly, effortful control may enhance the negative association between young children’s emotion regulation and CU behaviors.

Collectively, it can be inferred that effortful control may mitigate the impact of parental media soothing on children’s emotion regulation, while simultaneously enhancing the influence of emotion regulation on CU behaviors.

### 1.4. The Current Study

This study aims to examine the relationship between parental media soothing, children’s emotion regulation, and CU behaviors, as well as the interaction effects involving effortful control. Therefore, a hypothetical model was proposed and examined, including the following four hypotheses:

**H1.**  *Parental media soothing would significantly positively influence children’s CU behaviors.*

**H2.**  *Parental media soothing would correlate to children’s higher CU behaviors through the lower levels of emotion regulation.*

**H3.**  *Effortful control would moderate the relationship between parental media soothing and children’s emotion regulation.*

**H4.**  *Effortful control would moderate the relationship between children’s emotion regulation and CU behaviors.*

## 2. Methods

### 2.1. Participants and Procedure

A pilot test involving 43 parents was conducted before the main data collection to ensure the comprehension and active engagement with the survey content. Feedback from the parents indicated that the topics and scales were appropriate and of significant interest to them. Accordingly, the formal survey will include as many participants as possible to enhance effectiveness. Parents were recruited through convenience sampling. The significance of this study for child development was explained to the participants, and thus informed consent was received from parents who wished to gain relevant insights. Participants completed an anonymous online survey via the Wenjuanxing platform (accessed on 10 January 2025), the largest online network survey platform in China. The study protocol was approved by the research ethics committee of Guangzhou University (Protocol Number: GZHU202301).

In this study, a total of 1148 questionnaires were distributed, and data from 1095 parents were collected after excluding 53 invalid ones (e.g., missing some items, short duration, and obvious answer inconsistency), resulting in a high response rate of 95.38%. The age of their children ranges from 42 to 84 months (M_age(month)_ = 60.56, SD = 9.52), including 583 boys (53.2%, M_age(month)_ = 61.42, SD = 9.72) and 512 girls (46.8%, M_age(month)_ = 59.59, SD = 9.20). The demographic characteristics of the participating parents are shown in Table 1.

### 2.2. Measures

#### 2.2.1. Media Soothing

Parental media soothing was assessed using the Media Emotion Regulation Scale (Coyne et al., 2021). This 7-item scale, rated on a 5-point Likert scale (1 = never; 5 = always), evaluated parents’ use of media devices (e.g., smartphones, tablets, TVs) to regulate and calm children’s emotions and moods in contexts such as public outings, bedtime routines, or moments of upset. An example item is as follows: “After your child becomes upset, how often do you allow your child to watch a television show (on any device) to help him/her calm down?” Higher scores indicated a higher level of parental media soothing. In this study, psychometric analyses demonstrated strong internal consistency (Cronbach’s α = 0.90) and supported the construct validity through confirmatory factor analysis: *χ*^2^/*df* = 3.96, *p* < 0.001, CFI = 0.99, TLI = 0.98, RMSEA = 0.07 (90% CI [0.044, 0.089]), SRMR = 0.02.

#### 2.2.2. Emotion Regulation

Children’s emotion regulation was measured using the Emotion Regulation subscale from the Emotion Regulation Scale (Shields & Cicchetti, 1997), which has been validated for preschool children in the Chinese context (Zeng, 2020). This 5-item subscale measures children’s emotion regulation competence involving adaptive emotional expression, empathy, self-awareness, etc. Items (e.g., “He can express his feelings verbally when sad or angry”) were rated on a 4-point Likert scale (1 = never, 4 = always). A previous study has shown the great reliability and validity of this scale in Chinese preschool children (Tan et al., 2023). In the current study, Cronbach’s α of this scale was 0.93.

#### 2.2.3. CU Behaviors

The validated Chinese version of the Inventory of Callous–Unemotional Traits (ICU) was used to evaluate early CU behaviors (Frick, 2004; Deng et al., 2016). This scale consisted of 11 items measuring two core dimensions of uncaring (e.g., “He/she shows limited concern for others’ feelings”) and callousness (e.g., “He/she seems cold and inconsiderate”). The scores were reported on a 4-point Likert scale (0 = not true, 4 = definitely true). The higher the total score, the higher the degree of CU behaviors. Recent studies have indicated the great reliability and validity of this scale in Chinese preschool children (Zhu et al., 2024; Cao et al., 2023). In this study, the Cronbach’s α of CU behaviors was 0.86.

#### 2.2.4. Effortful Control

Children’s effortful control was assessed using the Effortful Control subscale of the Children’s Behavior Questionnaire-Very Short Form (Putnam & Rothbart, 2006; Allan et al., 2013). This 12-item scale is rated on a 7-point Likert scale (1 = completely inconsistent, 7 = completely consistent), including four dimensions: inhibitory control (e.g., the ability to delay gratification or inhibit inappropriate behaviors), attentional focusing (e.g., the ability to concentrate on tasks despite distractions), perceptual sensitivity (e.g., the ability to detect subtle changes in the environment), and low-intensity pleasure (e.g., the ability to derive enjoyment and satisfaction from calm, quiet, and low-stimulation activities). The short form of this scale has been validated to be applicable in the Chinese context (Zentner, 2020). The Cronbach’s α of the scale in the current study was 0.93.

### 2.3. Data Processing and Analysis

All statistical analyses of data were performed using SPSS 30.0 with the macro program (Hayes, 2013). First of all, to initially assess the potential presence of common method bias, Harman’s single factor test was conducted. Following that, to calculate descriptive statistics and bivariate correlations to evaluate relationships between key variables, this study constructed a Pearson correlation matrix including parental media soothing, children’s emotion regulation ability, CU behaviors, and effortful control. Thirdly, to further explore the indirect impact of children’s emotion regulation between parental media soothing and children’s CU behaviors and the interaction effects of effortful control, two macro-models were used in the further analysis. According to hypotheses 1 and 2, PROCESS Model 4 was performed to examine the indirect effect of emotion regulation on the link between parental media soothing and children’s CU behaviors. To verify hypotheses 3 and 4, Model 58 was used to verify the interaction effects of children’s effortful control.

## 3. Results

### 3.1. Testing for Common Method Bias

Principal component analysis (PCA) was employed for Harman’s single-factor test to assess potential common method bias (Podsakoff et al., 2024). After conducting a principal component analysis on all items, a total of seven factors with eigenvalues greater than 1 were extracted, explaining 59.30% of the variance. The first extracted common factor accounted for 23.99% of the variance, which is less than 40.0%, indicating that there is no significant common method bias.

### 3.2. Descriptive Statistics and Correlation Analysis of Variables

Pearson correlation analysis was used to examine the relationship between parental media soothing, children’s emotion regulation, CU behaviors, and effortful control. The means, standard deviations, and correlation matrices for each variable were shown in Table 2. The Pearson correlation coefficient revealed that media soothing had significantly negative associations with children’s emotion regulation (*r* = −0.17, *p* < 0.001) and effortful control (*r* = −0.20, *p* < 0.001), respectively. However, media soothing was positively correlated to children’s CU behaviors (*r* = 0.20, *p* < 0.001). Furthermore, emotion regulation was negatively associated with CU behaviors (*r* = −0.45, *p* < 0.001), while significantly and positively associated with effortful control (*r* = 0.51, *p* < 0.001). Effortful control had a negative association with CU behaviors actually (*r* = −0.42, *p* < 0.001).

In addition, children’s age was negatively associated with parental media soothing (*r* = −0.06, *p* < 0.05) and CU behaviors (*r* = −0.06, *p* < 0.05) and positively associated with effortful control (*r* = 0.08, *p* < 0.05). Children’s gender had significant associations with emotion regulation (*r* = −0.09, *p* < 0.01) and effortful control (*r* = −0.09, *p* < 0.01). Accordingly, age and gender would be included as covariates to exclude their effects in the subsequent analysis.

### 3.3. Testing the Indirect Effect Through Emotion Regulation

In order to reveal the indirect effect of children’s emotion regulation in the relationship between parental media soothing and early childhood CU behaviors, multiple regression was used, controlling for the effects of age and gender. The results showed parental media soothing could positively correlate to the increased CU behaviors in young children significantly (*β* = 0.20, *p* < 0.001), which supported hypothesis 1. Next, the PROCESS Model 4 was employed to construct a mediation model with children’s emotion regulation as a potential mediator. As shown in Table 3, the direct effect of parental media soothing on children’s CU behaviors was significant (direct effect = 0.06, *SE* = 0.01, Boot CI = [0.04, 0.09]), and the indirect effect of emotion regulation was significant as well (indirect effect = 0.04, *SE* = 0.007, Boot CI = [0.02, 0.05]). The indirect effect accounted for 36.57% of the total effect, significantly indicating the impact of parental media soothing on early CU behaviors through the decreased emotion regulation potentially, which supported hypothesis 2.

### 3.4. Testing the Difference in the Indirect Impact According to the Level of Effortful Control

As shown in Table 4, after controlling for the effects of children’s gender and age, the results showed that parental media soothing would positively be associated with the increasing of young children’s CU behaviors (*β* = 0.06, *p* < 0.001) but negatively impact emotion regulation (*β* = −0.05, *p* < 0.01). Then, CU behaviors could be negatively influenced by both emotion regulation (*β* = −0.20, *p* < 0.01) and effortful control (*β* = −0.10, *p* < 0.001) in young children. Conversely, effortful control significantly had a positive association with emotion regulation (*β* = 0.28, *p* < 0.001). Moreover, the interaction of parental media soothing and children’s effortful control could positively influence the developmental levels of emotion regulation (*β* = 0.08, *p* < 0.001), while the interaction of emotion regulation and effortful control would be associated with the decreasing of CU behaviors (*β* = −0.10, *p* < 0.001). Therefore, hypotheses 3 and 4 were supported, and effortful control acted as the moderator in the final model.

**Figure 1:**
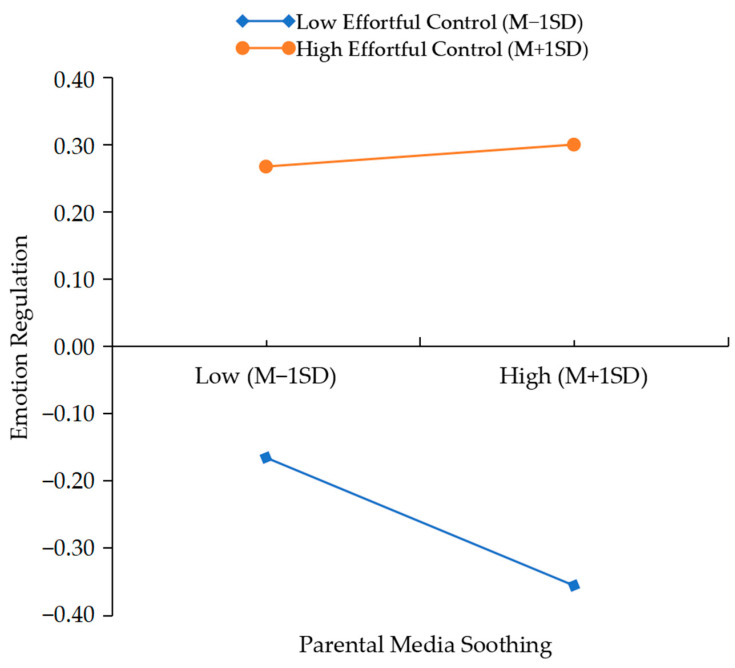
Interaction effect of PMS and children’s EC on ER.

**Figure 2:**
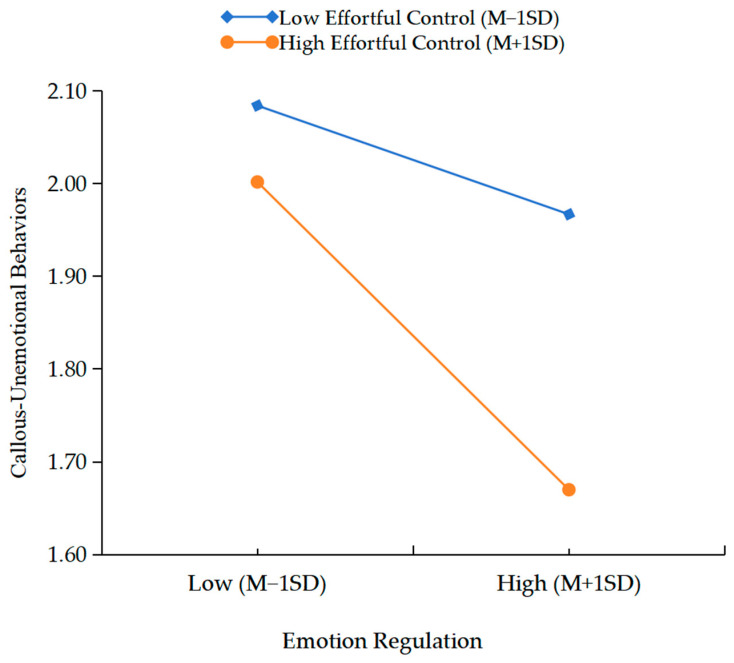
Interaction effect of children’s ER and EC on CU behaviors.

The indirect effect in the model under high and low levels of effortful control would be analyzed through adding and subtracting one standard deviation from children’s emotion regulation. As shown in Figure 1, for children with higher effortful control, parental media soothing could not significantly influence children’s emotion regulation (effect_higher_ = 0.02, *t* = 0.86, *p* = 0.39). However, as shown in Figure 2, the conditional indirect effect was significant in the lower effortful control (effect_lower_ = −0.13, *t* = −4.94, *p* < 0.001). Furthermore, children’s emotion regulation could be significantly related to the decreasing of CU behaviors both in the higher effortful control (effect_higher_ = −0.30, *t* = −12.63, *p* < 0.001) and the lower effortful control (effect_lower_= −0.11, *t* = −4.56, *p* < 0.001).

**Figure 3:**
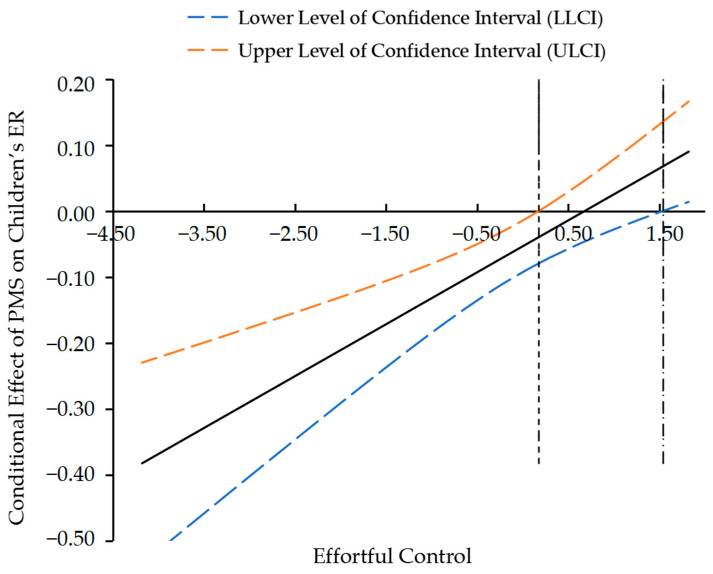
Johnson–Neyman plot of the moderating effect of EC between PMS and ER.

**Figure 4:**
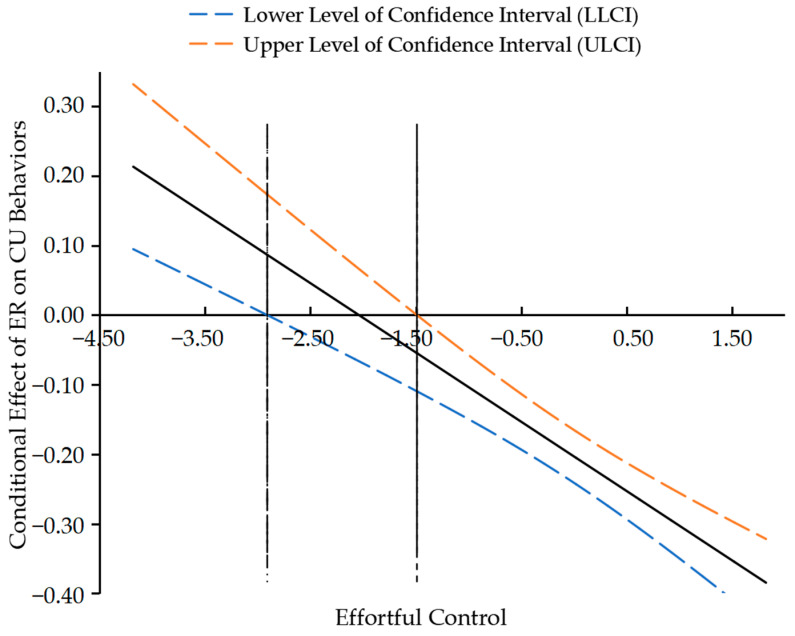
Johnson–Neyman plot of the moderating effect of EC between ER and CU behaviors.

Moreover, to precisely estimate the effect interval of children’s effortful control, the Johnson–Neyman technique was applied to analyze the significant intervals and cut-off scores. Firstly, Figure 3 indicated the influential role of parental media soothing on children’s emotion regulation reached statistical significance when the standard score of effortful control was less than 0.177 or greater than 1.542. Moreover, as shown in Figure 4, the influential role of children’s emotion regulation on CU behaviors was significant when the standard score of effortful control was less than −2.909 or greater than −1.489.

Thus, parental media soothing may promote the improvement of emotion regulation in children with higher effortful control but impede its development in those with lower effortful control. Similarly, emotion regulation significantly reduces the severity of CU behaviors in children with higher effortful control. However, for children with lower effortful control, especially below a certain threshold, the developmental risk of CU behaviors might be difficult to improve.

## 4. Discussion

The present study explored a moderated mediation effect within the theoretical framework between parental media soothing and children’s CU behaviors. The results showed that parental media soothing could positively impact the elevated levels of early CU behaviors significantly, and parental media soothing would correlate to the higher levels of children’s CU behaviors through the lower levels of emotion regulation. In addition, the indirect effect varies based on the levels of effortful control.

### 4.1. Parental Media Soothing Acts as the Potential Risk Factor for Early CU Behaviors

This study revealed that parental media soothing could significantly influence early CU behaviors positively, which is consistent with the finding of the recent study showing the negative association of parental media soothing and socioemotional outcomes in young children (Coyne et al., 2023). The present finding highlights the potential risks of relying on digital media as a primary strategy to manage children’s emotions. When parents use digital media to regulate and calm children’s moods, they might have fewer chances to talk about emotions with children. As a result, children may be less exposed to parental explanations and strategies for dealing with emotion reactivity, which subsequently leads to their children’s limited prosocial emotions (Coyne et al., 2023).

In other words, parental media soothing may make children become callous and indifferent in interpersonal interactions, because children may be used to the direct comfort provided by electronic devices. In addition, screens would foster callousness. The media use of young children would replace the necessary communication and social exchange between children and parents, thereby affecting the development of secure attachment (Linder et al., 2021). The warm interaction and parental comforting are closely related to parent–child attachment (Q. Liu & Wang, 2021). However, the engagement of media during children’s distress would further reduce the opportunities to form a secure attachment with their parents, lacking the emotional engagement and social learning inherent in face-to-face interactions (Radesky et al., 2016). Over time, the reliance on external media devices undermines children’s adaptive socioemotional outcomes, such as deficits in empathy, shame, and emotional functioning (Coyne et al., 2023). Consequently, parental media soothing is positively related to the severity of CU behaviors in Chinese preschool children.

However, causality cannot be established due to the cross-sectional design, which does not account for the possibility that parents may use media in response to CU behaviors. Moreover, it is significant to acknowledge that the relationship between digital media use and children’s CU behaviors may be impacted by underlying social determinants of health (e.g., socioeconomic stress, access to resources) and caregiver well-being (Spencer et al., 2022), which might shape parental capacity and choices.

### 4.2. The Indirect Impact Through Children’s Emotion Regulation Ability Potentially

The results indicated that emotion regulation plays a potential mediator between parental media soothing and children’s CU behaviors. Specifically, it is suggested that parental media soothing could correlate with the severity of CU behaviors through weakening children’s adaptive emotion regulation. Notably, the recent literature held that for children experiencing negative emotion reactivity, parental deployment of digital devices to distract children’s attention would help to regulate their emotions (Snyder et al., 2025). A recent study showed that active media exposure in Chinese preschoolers was positively correlated to emotion regulation, but on the condition of high-quality caregiver companionship (Wu et al., 2023), rather than using digital devices to calm children down alone. When parents frequently conduct media-mediated regulation, it might reduce the quality of parent–child interactions and parental verbal comfort directed at young children (Anderson & Hanson, 2017), hindering children’s ability to autonomously recognize emotions and practice self-regulation strategies, thereby depriving them of critical opportunities to acquire emotional management skills (Coyne et al., 2021). For Chinese parents, the boundaries of such parenting practices in terms of digital media may be quite ambiguous, thus potentially increasing the occurrence of related negative impacts on children, likely CU behaviors.

Additionally, the previous study has shown that emotion regulation is seen as the key factor of CU behaviors (Young & Kyranides, 2022; Mihic & Novak, 2018). Children with emotion dysregulation generally show emotional impulsiveness, instability, and weakened emotional responses to negative stimuli (Paulus et al., 2021). Over time, children’s emotion dysregulation might lead to the emergence of empathy deficits and CU symptoms (Truedsson et al., 2019). Similar to the findings of the existing study (Wilke et al., 2025), children with stronger emotion regulation abilities would show reduced CU behaviors, while those with weaker skills are at higher risk for increased CU behaviors. Accordingly, parental media soothing would be associated with the elevated levels of early childhood CU behaviors by inhibiting the acquirement of emotion regulation ability. Thus, parents should seriously monitor the selected contents and frequency when using media to soothe their children’s emotions (Snyder et al., 2025). Moreover, parents are encouraged to co-use media with children to conveniently change children’s cognitive appraisals and response modulation (Gross, 2014).

### 4.3. Effortful Control Matters Significantly for Early Socioemotional Development

Effortful control, as an internal protective factor, could mitigate the adverse effects of parental media soothing on children’s emotion regulation, in turn indirectly reducing the risk of CU reactions. The existing evidence showed that effortful control could help individuals regulate emotional responses through the executive attention mechanisms (Simonds et al., 2007). Children with higher effortful control might be less likely to rely on their parents for media-based emotion regulation and soothing (Elizur et al., 2017). In some cases, these children may even proactively advise their parents to limit smartphone use. In other words, children with higher effortful control could show greater psychological resilience, enhanced inhibition of impulsive responses, and maintained concentration (Rothbart et al., 2009), which enabled them to develop adaptive emotion regulation strategies even when exposed to media screens and negative parent–child joint media engagement.

In contrast, children with lower effortful control would experience more negative impacts of situational risks than those with higher effortful control (Lengua et al., 2008). A recent study has indeed found that parental media soothing could be beneficial for children’s negative emotionality (Snyder et al., 2025). However, for young children with low effortful control, the negative impact of parental media soothing on emotion regulation might be more pronounced. Meanwhile, some parents may increasingly turn to digital media to regulate and manage their children’s emotional states. However, whether this becomes a risk also partly depends on the child’s temperament, such as effortful control. Thus, it is inferred that children with lower effortful control might have difficulty in disengaging from media-based emotion coping and are prone to addiction to media devices. Therefore, for children with low effortful control, parents should limit digital media involvement to avoid possible addiction to digital devices. Meanwhile, parents could offer tangible emotional support and engage in emotion-focused conversations to boost children’s self-efficacy in regulating emotions and impulse reactions and to prevent the emergence of CU behaviors.

In addition, children’s effortful control would amplify the protective effect of emotion regulation against CU behaviors, which highlights the role of effortful control in strengthening the positive adaptation pathway (Graziano et al., 2022). For instance, effortful control of young children could moderate the influential degree of negative emotionality on externalizing symptoms and socially inappropriate behaviors (Wilson et al., 2021). Similarly, this present finding corroborates the protective–protective model (Fergus & Zimmerman, 2005), in which a high level of effortful control might enhance the protective effect of emotional flexibility and facilitate better regulation of emotions, thereby reducing the risk of developing CU behaviors in early childhood.

## 5. Conclusions and Limitations

CU behaviors have increasingly been recognized as a key indicator of early socioemotional maladjustment and behavioral problems. The current study explored the association of the occurrence of early CU behaviors and parenting practices that regulate children’s negative emotionality through digital media and highlighted the significance of recognizing both the convenience and the risks related to digital media in the parenting process. Parental media soothing significantly impacted early childhood CU behaviors, with the indirect roles of children’s emotion regulation and effortful control within this model.

Nevertheless, there are several limitations of this study. First, although noteworthy effects were identified, the cross-sectional approach could not establish causal relationships and reciprocal influences among variables. Second, while focusing on parental media soothing, contextual factors associated with this emerging parental practice and early CU behaviors remain insufficiently examined. For example, the content and application of digital media, as well as parental motivations for media soothing, could not be adequately considered in this study.

Therefore, based on the present findings, future research should further explore reciprocal effects among parental media soothing, CU behaviors, and other variables such as the structural factors using longitudinal designs. Additionally, the dynamics of parental media soothing and children’s emotional states could be examined through observational data in future fieldwork. If possible, teachers might be invited to report on children’s CU behaviors in the classroom. This multi-informant approach might broaden the perspectives in future studies.

## Figures and Tables

**Table 1:** Demographic characteristics of the parents.

Variables	Category	Frequency	Percent (%)
Gender	Male	528	48.2
	Female	567	51.8
Place of Residence	Urban	1022	93.3
	Rural	73	6.7
Monthly Income (CNY)	≤2000	154	14.1
	2001–4000	241	22.0
	4001–7000	361	33.4
	7001–10,000	193	17.6
	≥10,001	141	12.9
Educational Levels	Junior High School and Below	119	10.9
	High School and Technical Secondary School	190	17.3
	Junior College	299	27.3
	Undergraduate	418	38.2
	Postgraduate and Above	69	6.3
Professions	Freelance	153	14.0
	Farmers	15	1.4
	Businessmen	200	18.3
	Teacher/Doctor	177	16.2
	Workers	314	28.6
	Officers	236	21.5

**Table 2:** Descriptive statistics and correlations between measured variables.

Variables	*M*	*SD*	1	2	3	4	5	6
1. Child age (month)	60.56	9.52	—					
2. Gender	0.53	0.50	0.10 **	—				
3. Parental media soothing	2.07	0.73	−0.06 *	0.03	—			
4. Emotion regulation	3.08	0.56	0.03	−0.09 **	−0.17 ***	—		
5. CU behaviors	1.90	0.36	−0.06 *	0.05	0.20 ***	−0.45 ***	—	
6. Effortful control	5.18	0.97	0.08 *	−0.09 **	−0.20 ***	0.51 ***	−0.42 ***	—

N = 1095. Gender was a dummy coding variable. Boy = 1. Girl = 0. * *p* < 0.05. ** *p* < 0.01. *** *p* < 0.001.

**Table 3:** Analysis of direct effects in the mediation model.

	Emotion Regulation				CU Behaviors			
	*β*	*SE*	*t*	95% *CI*	*β*	*SE*	*t*	95% *CI*
Parental media soothing	−0.13	0.02	−5.68 **	[−0.17, −0.08]	0.06	0.01	4.58 ***	[0.04, 0.09]
Emotion regulation					−0.28	0.02	−15.59 ***	[−0.31, −0.24]
Age (month)	0.002	0.002	1.07	[−0.002, 0.005]	−0.002	0.001	−1.48	[−0.004, 0.001]
Gender	0.10	0.03	3.09 **	[0.04, 0.17]	−0.006	0.02	−0.33	[−0.05, 0.03]
*R* ^2^	0.04				0.22			
*F*	14.69 ***				76.35 ***			

N = 1095. ** *p* < 0.01. *** *p* < 0.001.

**Table 4:** Evaluating the moderated mediation effects: EC as the moderator.

	Emotion Regulation				CU Behaviors			
	*β*	*SE*	*t*	95% *CI*	*β*	*SE*	*t*	95% *CI*
Parental media soothing	−0.05	0.02	−2.69 **	[−0.09, −0.02]	0.06	0.01	4.50 ***	[0.03, 0.08]
Emotion regulation					−0.20	0.02	−10.51 **	[−0.24, −0.17]
Effortful control	0.28	0.02	18.36 ***	[0.248, 0.307]	−0.10	0.01	−8.72 ***	[−0.12, −0.08]
Parental media soothing × Effortful control	0.08	0.02	4.36 ***	[0.043, 0.114]				
Emotion regulation × Effortful control					−0.10	0.01	−7.22 ***	[−0.13, −0.07]
Age (month)	0.000	0.002	−0.003	[−0.003, 0.003]	−0.001	0.001	−0.68	[−0.003, 0.001]
Gender	0.05	0.03	1.77	[−0.006, 0.108]	0.01	0.02	0.58	[−0.03, 0.05]
*R* ^2^	0.28				0.29			
*F*	85.83 ***				75.47 ***			

N = 1095. ** *p* < 0.01. *** *p* < 0.001.

## Data Availability

The original contributions presented in this study are included in the article. Further inquiries can be directed to the corresponding author(s).
